# Draft Genome Sequence of Streptomyces mexicanus Strain Q0842, Isolated from Human Skin

**DOI:** 10.1128/MRA.01527-19

**Published:** 2020-10-29

**Authors:** Manon Boxberger, Mariem Ben Khedher, Anthony Levasseur, Bernard La Scola

**Affiliations:** a Aix Marseille Université, IRD, AP-HM, MEФI, Marseille, France; b IHU-Méditerranée Infection, Marseille, France; University of Maryland School of Medicine

## Abstract

In 2003, *Streptomyces mexicanus* was reported as a novel xylanolytic bacterial species isolated from soil; a partial genome sequence was determined. In 2019, a strain from the same species was isolated from a hand skin swab sample from a healthy French woman. Genome sequencing revealed an 8,011,832-bp sequence with a GC content of 72.5%.

## ANNOUNCEMENT

In 2003, Petrosyan et al. described a novel bacterial species within the family *Streptomycetaceae,* in the phylum *Actinobacteria*, named Streptomyces mexicanus (1). The *Streptomyces* genus contains 856 species with validly published names (2). In 2019, we isolated strain Marseille-Q0843 from a hand skin swab sample from a healthy French woman using the culturomics approach (3–5) with the aim of identifying the skin flora. The study was approved by the CPP-Sud Méditerrannée IV ethics committee (IDRCB 2019-A01508-49). Identification based on 16S rRNA gene sequencing showed 99.04% similarity to Streptomyces mexicanus strain CH-M-1035^T^ (GenBank accession number AF441168.1). Our strain was initially isolated by direct seeding of 50 μl of sample, and growth was observed after 24 h on 5% sheep blood-enriched Columbia agar (bioMérieux, Marcy l’Etoile, France) under aerobic conditions at 31°C. The strain was then routinely cultivated under the same conditions. In 2014, a partial genome (2,089,349 bp) of the Streptomyces mexicanus type strain JCM 12681 became accessible (GenBank accession number BBGQ01000001.1). Here, we propose the most complete genome sequence of *S. mexicanus*.

Genomic DNA (gDNA) was extracted on the BioRobot EZ1 system (Qiagen, Hilden, Germany) with the EZ1 DNA tissue kit (Qiagen) according to the manufacturer’s instructions, quantified by a Qubit assay with the high-sensitivity kit (Life Technologies, Carlsbad, CA, USA) and adjusted to 0.2 ng/μl, and sequenced on the MiSeq platform (Illumina, Inc., San Diego, CA, USA) with the paired-end strategy after preparation with the Nextera XT DNA sample preparation kit (Illumina). Subsequently, 12 cycles of PCR amplification completed the tag adapters and introduced dual-index barcodes. After purification on AMPure XP beads (Beckman Coulter, Inc., Fullerton, CA, USA), the libraries were normalized on specific beads according to the Nextera XT protocol (Illumina). Total information of 14.8 Gb was obtained from a cluster density of 763,000 clusters/mm^2^, with clusters passing quality control filters at 96.2%. Within that run, the index representation for *S. mexicanus* strain Marseille-Q0842 was determined to be 6.64%. The 1,895,038 paired-end reads were filtered according to the read qualities with Trimmomatic v0.36 software (6) with default parameters. To improve the quality of the sequence, the Oxford Nanopore Technologies (ONT) approach was used for the same gDNA extract, with 1D gDNA sequencing on the MinION system using the SQK-LSK109 kit. A library was constructed from 1.5 μg gDNA without fragmentation and end repair. Adapters were ligated to both ends of the gDNA. A total of 1,409 active pores were detected for the sequencing, and the workflow WIMP was chosen for bioinformatic analysis. After the 2-h run time and the end of the life of the flow cell, 164,860 raw reads were generated. The *N*_50_ for the ONT reads is 7,315 nucleotides.

**FIG 1:**
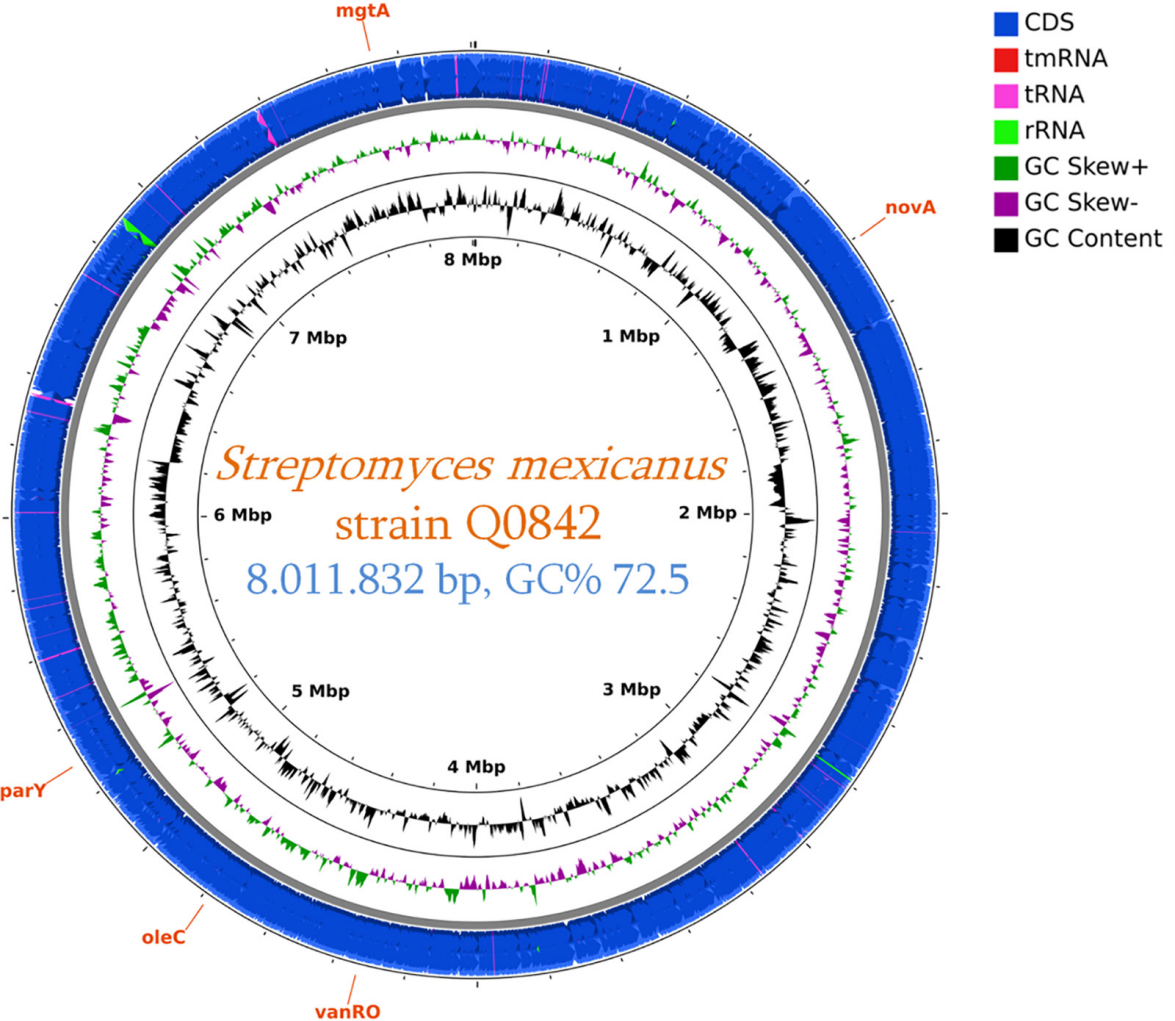
Circular genome map for *Streptomyces mexicanus* Marseille-Q0842, generated using CGView (11). The following features are shown (moving from the outermost track inward, with the origin of replication positioned at 0 kbp): CDSs (blue), tRNAs (pink), and rRNAs (light green), positive and negative GC content skew (green and purple, respectively), GC content (black), and genome position. The resistance genes (orange) were identified by the CARD database.

Assembly of the genome was performed, using the data obtained by both sequencing methods, by SPAdes v3.14.1 software (7) and was manually finished by using sequence similarity searches and blocks conserved between closest species in the *Streptomyces* genus. The genome includes 30 contigs (with an *N*_50_ value of 475,868 bp) and consists of 8,011,832 bp with a GC content of 72.5%. Genome annotation was obtained through the NCBI Prokaryotic Genome Annotation Pipeline (PGAP) v4.12 (8). Of the 7,006 predicted genes, 6,546 were protein-coding genes, 88 were RNAs (3 noncoding RNAs [ncRNAs], 6 5S rRNAs, 6 16S rRNAs, 6 23S rRNAs, and 67 tRNAs), and 372 were pseudogenes. The *in silico* resistome of this strain was obtained by using the CARD v3.0.7 database (9), and the search for virulence factors was performed by using the Virulence Factor Database (10). Default parameters were used for all software unless otherwise specified. An overview of the genome features, including coding sequences (CDSs), rRNAs, tRNAs, and resistance genes identified, is shown in Fig. 1.

### Data availability.

The genome and reads for Streptomyces mexicanus strain Marseille-Q0842 have been deposited in GenBank under the accession numbers JACMHY000000000.1, ERR3721841 (Illumina reads), and PRJNA647647 (ONT reads), respectively.
